# Sir John Struthers, M. D., LL. D.

**Published:** 1899-03

**Authors:** 


SIR JOHN STRUTHERS, M. D., LL. D., vice-president of the Royal College of Surgeons, Edinburgh, and examiner in anatomy Royal College of Surgeons, died February 24, 1899.

Sir John was born at Brucefield, Scotland, on February 21, 1823, and was graduated in medicine from Edinburgh University in 1845. He immediately became a demonstrator in anatomy at the university, and was lecturer on that subject from 1847 to 1863. He was also examiner in the College of Surgeons, and surgeon to the Royal Infirmary. In 1863 he was appointed professor of anatomy in the University of Aberdeen, and held that post until 1889, when he retired from teaching. He formed an extensive museum of human and comparative anatomy for Aberdeen. He took an active part in the questions of medical and university reform, which were settled by the medical act of 1886 and the Scottish Universities act of 1889.

In 1885 the University of Glasgow conferred on him the degree of LL. D. He was knighted in 1898. Sir John published numerous articles on subjects connected with human and comparative anatomy in the Edinburgh *Medical Journal* and in *The Journal of Anatomy and Physiology*, and also several separate works, among which were a historical sketch of the Edinburgh Anatomical School.

